# Is there a civic duty to support medical AI development by sharing electronic health records?

**DOI:** 10.1186/s12910-022-00871-z

**Published:** 2022-12-10

**Authors:** Sebastian Müller

**Affiliations:** Center for Life Ethics/Heinrich Hertz Chair TRA4, University of Bonn, Schaumburg- Lippe-Straße 5-7, 53113 Bonn, Germany

**Keywords:** Artificial Intelligence, Public Health, Informed consent, Biomedical Research, Health data, Ownership, Meta consent

## Abstract

Medical artificial intelligence (AI) is considered to be one of the most important assets for the future of innovative individual and public health care. To develop innovative medical AI, it is necessary to repurpose data that are primarily generated in and for the health care context. Usually, health data can only be put to a secondary use if data subjects provide their informed consent (IC). This regulation, however, is believed to slow down or even prevent vital medical research, including AI development. For this reason, a number of scholars advocate a moral civic duty to share electronic health records (EHRs) that overrides IC requirements in certain contexts. In the medical AI context, the common arguments for such a duty have not been subjected to a comprehensive challenge. This article sheds light on the correlation between two normative discourses concerning informed consent for secondary health record use and the development and use of medical AI. There are three main arguments in favour of a civic duty to support certain developments in medical AI by sharing EHRs: the ‘rule to rescue argument’, the ‘low risks, high benefits argument’, and the ‘property rights argument’. This article critiques all three arguments because they either derive a civic duty from premises that do not apply to the medical AI context, or they rely on inappropriate analogies, or they ignore significant risks entailed by the EHR sharing process and the use of medical AI. Given this result, the article proposes an alternative civic responsibility approach that can attribute different responsibilities to different social groups and individuals and that can contextualise those responsibilities for the purpose of medical AI development.

## Background

In 2017, Stanford University AI *CheXNet* diagnosed pneumonia more accurately in a test run than three out of four competing human radiologists. How did *CheXNet* learn to do this? In a supervised training process, the algorithm accessed a database containing chest X-ray images, with each image linked to diagnostic metadata. The algorithm used the labelled data to extract new recognition patterns for X-ray diagnostics [1]. *CheXNet* is one of many examples of artificial intelligence (AI) in medicine. Medical AI is used to improve diagnostics, therapies, nursing practices, preventive care, and emergency medicine [2–4]. It can be used to predict a healthy person’s risk of falling ill or a sick person’s risk of dying [5]. Medical AI can also be used to optimise medical research, patient management, health care systems, and drug development [6, 7]. The various application possibilities underline the algorithms potential benefits. To accomplish their tasks, medical AIs need to access and process different types of training data i.a. data sets that are used by machine learning algorithms to optimise their modelling abilities. *CheXNet*, for example, trained its diagnostic abilities with the help of ChestX-Ray14^1^, a publicly available database. This database contains over 110,000 chest X-ray images and corresponding diagnostic records from 30,805 individuals. Multiple other public and private databases alike contain unique compositions of data.

In most cases, the data that are used to train medical AI are not generated for the purpose of medical AI development. Instead, data is extracted from personal electronic health records (EHRs)^2^, that is, digitalised health data that is generated in the process of the medical treatment of a human being. EHRs can include a patient’s medical history, diagnoses, treatment plans, immunisation dates, allergies, medications, image data, laboratory test results and other factors [10, 11]. Since these data are considered particularly sensitive (e.g., EU Commission Regulation 2016/679), they can only be approved for reuse in medical AI development projects under certain conditions: the EHRs are completely anonymised, or a governmental act is in place (e.g., reporting of COVID-19 cases), or data subjects have provided their informed consent (IC). IC means that human beings are informed about the purpose of the secondary use of their health data and the associated risks and benefits before they choose to give their consent or refusal. Since the consent process is associated with a disproportionately higher administrative burden and a lower quantity of training data compared to anonymised and IC-free AI development processes, the latter is often preferred [12, 13].

However, many medical AI projects rely on identifiable or pseudonymised training data. In addition, there is the risk of re-identification of anonymised or pseudonymised health data, which increases as AI technology grows more sophisticated [14–16]. One example of such a re-identification process is the deep learning algorithm developed by Packhäuser and colleagues, which can link anonymised images from the already mentioned *ChestX-Ray 14* database to non-anonymised chest X-rays in other data collections [17]. With AI technology that is able to recognise and link faces, fingerprints, voices, or genetic material the issue of re-identification becomes more relevant [18]. As such, many medical AI development projects today rely on people providing IC to use their EHRs.

According to numerous scholars, the privilege of individual data subjects to affect secondary EHR use in medical data research via their IC decisions separates today’s medical care from the innovative health care of the future [19–23]. To pave the way to this future, a number of scholars advocate a moral duty for citizens to share their EHRs for medical research [19, 22–25]. In this context, the term duty means that all citizens who do not engage in EHR sharing are morally blameworthy. Certain scholars even believe this duty to be so imperative that it might be used to bypass IC in some circumstances [22, 24]. Even though they are not always framed as such, the arguments in favour of a duty to support medical research and innovations by sharing EHRs pertain to people in their social role as citizens. They do so because they rely on normative constructs such as solidarity, mutual socio-political recognition, and culturally shared ideals of justice that presuppose a concept of citizenship.

To the best of the author’s knowledge, there are three dominant lines of reasoning in favour of this duty: citizens have a moral duty to share their EHRs to support medical research if (i) doing so conforms to the rule to rescue, if (ii) it is beneficial for society and only involves low personal risks, and/or if (iii) the EHR in question can be considered a good for which citizens cannot claim exclusive property rights. It is interesting that the advocates of these arguments do not address medical AI in particular, even though the normative framework of their arguments considers this technology. Thus, the normative issues associated with medical AI research, development, and use are not considered in the discussion.

This article will refute all three propositions in the context of medical AI development and show that there are only a very small number of cases imaginable in which it is justified to call for the civic duty in question. It will also be argued that in all other cases, it is more fruitful to talk about a moral responsibility to share certain types of data under certain conditions for the development of certain types of medical AI.

## Discussion

### Medical AI and the civic duty to share EHRs

Two noteworthy articles by Ploug [26] and Ballantyne [27] identify the most discussed arguments in favour of a civic duty to share EHRs for medical research. The articles focus on three main arguments. The first argument, which I call the ‘rule to rescue argument’, states that there is a universal rule to rescue people in accident-like situations and that people should conform to this rule by sharing EHRs [22, 23, 28]. The rule implies a duty to support medical AI developments that can rescue people in accident-like situations. Second, there is the ‘low risks, high benefits argument’, which says that people have a duty to benefit others as long as the risks entailed by doing so are bearable [23–25, 29]. If a medical AI development project complies with this risk-benefit ratio, there is a civic duty to share EHRs. The third argument, the ‘property right argument’, emphasises that a great deal of health data is not generated by private citizens but collected and distributed within the health care process. Advocates of this argument believe that since such processes are mostly financed by solidarity-based health care contributions and taxpayers’ money, the results of those processes are a public good to which citizens should not claim exclusive rights [21, 22, 30]. Since medical AI development is an important part of modern medical research, and since none of the advocates of a civic duty to share EHRs excludes medical AI from their discussion, I will assume that the three arguments in favour of a civic duty to support medical research includes sharing EHRs with medical AI research and development projects.

### The ‘rule to rescue argument’

The *rule to rescue* is a well-known bioethical imperative that imposes a duty to prevent harm from happening to other people [26, 31, 32]. Following that rule, an entity A_i_ has a duty to rescue a human being B_i_ in an accident-like situation µ_i_ if and only if (i) A_i_ is part of the situation, if (ii) A_i_ is able to provide proper help in µ_i_, if (iii) there is no other entity A_n ≠ i_ that is more capable of providing help in µ_i_, and if (iv) compliance with the rule does not force A_i_ to sacrifice anything of equally important moral value. A situation µ_i_ can be characterised as accident-like if there is a high risk of significant loss of or damage to B_i_’s basic interests and if those risks can only be prevented by the immediate action(s) of others.

What moral obligations does this rule imply in practice? Witnesses of a car crash have a duty to call emergency services, physicians that witness a passenger on an airplane having a heart attack can be expected to render first aid, and a fishing company has a duty to save its employees when the engine of one of their ships breaks down at sea.

As Rulli and Millum have discussed, the rule and its application become more complex when collective actors are involved [28]. There are many accident-like situations µ_i_ in which victim B_i_ cannot be rescued by one person but only by a group of people. For example, the person who witnesses an accident is usually considered to have a duty to call an ambulance. Emergency services are usually considered to have a duty to aid the victim and take her or him to the emergency room (ER), the physician on duty is expected to provide medical care, and the institutions that are involved in the rescue process are expected to allocate resources, write laws, and accumulate knowledge in a way that promotes such care. The social roles of the people involved in such processes determines the scope and force of their duty [33, 34]. The ER physician, for example, has a duty to rescue a patient who is being taken to the ER. The same physician, however, does not have a specific duty to rescue people from a burning house. This task is the professional duty of firefighters. Therefore, the *rule to rescue* is not shaped by the endangerment of B_i_’s basic interests but by the social context in play and the type of entity that A_i_ is. The rule can call for individual duties such as the duty to call an ambulance, social role duties such as the duty of an ER physician to heal a patient, and institutional duties such as the duty to properly fund emergency services [28, 35].

A number of scholars believe that the *rule to rescue* implies a personal moral duty to support medical research by sharing EHRs [22, 23, 30, 36]. The argument is as follows: Physicians in ERs, paramedics, and others save people’s lives on a daily basis. Because certain innovative health care practices, innovative medical technologies and forms of advanced medical knowledge are tools that enable people (A_i_) to rescue others (B_i_) in accident-like situations (µ_i_), people have a duty to support such research and, thus, such technological developments. Typically, this duty is considered to be a subset of the bioethical duty of beneficence. Because certain medical AIs can be used in medical emergencies [37] the development of these AIs need be considered by the *rule to rescue* argument. The argument’s structure can be broken down as follows:

1. Certain types of medical AI can rescue human beings in accident-like situations.
2. Citizens have a moral duty to conform to the *rule to rescue.*
3. The development and improvement of medical AI that can rescue human beings in accident-like situations requires EHRs.
4. Citizens have a moral duty to support medical AI developments that can rescue human beings in accident-like situations by sharing EHRs.

#### Why the rule to rescue does not apply in the medical AI context

Examining this argument closely, P1 and P3 appears to conflict with one another. Ploug notes [26] that no victim B_i_ has ever been rescued by the act of EHRs sharing. Instead, a victim is rescued by another person that might or might not use technology that was developed in the past. This temporal shift between data sharing and the rescue act is highly relevant for the ‘rule to rescue argument’ because there are no urgent situations µ_i_ in which a passer-by can provide proper help to a victim B_i_ simply by sharing her EHRs. The sharing component necessarily precedes the entire rescue scenario, which means that the entity A_i_ that performs the rescue is not the same as the entity C_i_ that shares her data. I think Plougs critique applies to medical AI development as well. Take any accident-like scenario in which a medical AI supports a rescue process. For example, a clinical decision support system may enable a physician to save a patient’s life because it recognises indicators for a rare disease. In this and all other scenarios, the victim is rescued either by an automatous algorithm that was trained with EHRs before the rescue took place or by another person who uses medical AI as a tool.

One might now argue alongside Rulli and Millum [28] that within the *rule to rescue*, it is possible to distinguish among different sets of duties. For example, to protect citizens in a pandemic, politicians and scientific consultants have the professional duty to introduce effective preventive measures. If the chances of selecting the right combination of preventive measures can be increased with the help of a medical AI and its access to EHRs, politicians and consultants may have a professional duty to use those tools. Intertwined with that duty is the institutional duty of supporting everyone employed in the rescue process with sufficient tools and resources. This institutional duty may be composed of many other duties, such as the professional duty of researchers to develop medical AI that can simulate pandemics and the effect of different preventive measures, the federal duty to financially support such research, and the civic duty to grant access to the data that is needed to develop the AI and run the simulations. Even if this line or argumentation were accepted, there would still be the problem that an institutional duty to rescue those in peril cannot imply a civic duty to share EHRs without further ado. Additional arguments are needed to explain how exactly an institutional duty towards the *rule to rescue* can have an impact at the level of individual citizens and why it gives rise to a duty to share EHRs rather than other responsibilities. Without further references to normative values such as beneficence and justice, normative trade-off rationales, or theories concerning collective responsibility, citizens might as well live up to the *rule to rescue* by paying their taxes, donating blood, advocating better research conditions, or conforming to well-known preventive health measures. Because the individual act of data sharing does not cause anybody to be rescued, and because a call for an institutional interpretation of the *rule to rescue* does not entail an imperative to share EHRs, the ‘rule to rescue argument’ cannot justify a duty to support medical AI developments that will save people in accident-like situations.

### The ‘low risks, high benefits argument’

The principle of beneficence is a positive requirement to promote the welfare of others and contribute to the common good [38]. In medical contexts, this principle obliges caregivers and researchers to act in accordance with the interests of their patients and research subjects. In business, it obliges companies to conduct their business in a way that serves social interests. In democratic politics, it obliges citizens to vote and act in ways that increase and foster the common good. The principle of beneficence is often supplemented by concepts of solidarity and justice that advocate (i) a duty to act in ways that benefit the members of a given society, including oneself [39, 40], and (ii) social structures that promote equality [20, 30].

Scholars that support the ‘low risk, high benefit argument’ link the principle of beneficence with the belief that all citizens of modern societies will benefit significantly from innovative health care developments. The overall benefits are or will be so significant, they argue, that the risks associated with EHRs sharing are negligible in comparison. Therefore, citizens have a moral duty to share EHRs to support medical research [23–25, 29].

It is necessary to take a closer look at the benefits and risks that the authors present to discuss the argument sufficiently. And since medical AI is part of medical research and can be used to promote medical and social health benefits [12], I will also consider medical AI specific risks and benefits. Let’s start with the benefits. Schaefer and colleagues for example point to the potential improvement in public health care and personal well-being to justify the moral duty to share EHRs for medical research [29]. Bowten and colleagues add the decrease of health care costs to this list [41] and Knottnernus points to the benefits that large EHRs databases provide for the expansion of medical knowledge [21].

By focusing on the medical AI development, I believe a further benefit needs to be mentioned. Forsberg and colleagues claim that all citizens will receive substantially worse health care in the future compared to an ideal scenario if some citizens do not support key innovations such as medical AI technology today [24]. This argument is quite strong, as it relates to issues of discrimination and injustice surrounding so-called *selection bias*. Selection bias can occur when an AI is trained with datasets in which groups of a certain age, social class, ethnicity, biomarker, or health status are underrepresented or unrepresented [42, 43]. An AI that is biased in that respect might not be able to recognise signs of skin cancer on a skin tone with which it is unfamiliar, it might diagnose women less accurately than men if it is primarily trained with male data, and it may not recognise certain cases of dangerous drug interactions if it is denied access to the data of vulnerable groups like Alzheimer patients [44]. According to a review study by Kho and colleagues, selection bias is not the result of a few citizens who refuse to share EHRs but of an effect called *consent bias*, which means that certain socio-economic groups are structurally more willing to consent to health-related research than others [45]. Selection bias might also be affected groups that generate more useful data than others, such as chronically ill people or quantified self-enthusiasts [46]. Since medical AIs are only as good as training data allow them to be, Cassell and Young call for a duty to foster a balanced representation of all social groups in those data [25]. This proposition means that people who belong to groups that are underrepresented in medical datasets, which in most cases is everyone except adult Caucasian men [47], have a specific civic duty to share their EHRs.

Now what about the minimal or reasonable risks that come along with EHR sharing, and the risks entailed by the development and use of medical AI? Minimal risks are risks that are perceived as normal in everyday encounters such as driving a car or going to the dentist for a routine check-up [48]. The risks associated with EHR sharing and the development and use of medical AI can be of very different natures and can affect individuals, social groups and institutions differently [49]. Individuals can suffer personal harm due to data breaches caused by hacker attacks, data misuse, or adversarial attacks [22, 50]. Individuals can also be harmed by medical AIs that produce technical errors, give wrong medication advice or misinterpret input data [51]. Social groups can be discriminated against and treated unjustly by biased AI [52], and institutions such as health care professions can be severely harmed if people develop trust issues and avoid medical treatment [53]. Unfortunately, it is hard to decide whether those risks are comparable to the risks of other everyday activities and, therefore, qualify as minimal. It is also hard to decide whether those risks are small in comparison to the benefits and, therefore, reasonable. A strategy to solve this problem is to empirically prove that certain risks, such as the personal risk of harm from EHR breaches, are statistically smaller than other everyday risks, such as the personal risk of harm from traffic. Porsdam-Mann and colleagues [23] proceed with this strategy and estimate that the personal risk of becoming a victim of health data-related privacy breaches in the US was approximately 0.02%^3^ between 2009 and 2016. In contrast, the risk of being injured in traffic in 2009 was approximately 0.7%. Considering these risks, the authors conclude that researchers should be allowed to access at least low risk datasets without asking for IC.

Based on the risk-benefit analysis presented and the presupposed principle of beneficence, the ‘low risk, high benefit argument’ is:

1. Citizens have a moral duty to benefit others.
2. The risks of developing and using such medical AI are reasonable.
3. The development and use of certain forms of medical AI is beneficial for society.
4. The development and improvement of medical AI requires EHRs use.
5. Citizens have a moral duty to support medical AI developments that can be expected to benefit society by sharing EHRs.

#### Why the risks of medical AI development and the use of such AI can be unreasonably high

For analysis of premise P2, it is vital to acknowledge the two interrelated notions of risk as the quantifiable probability of a harm being done and as the quality of a harm. As mentioned previously, some works focus exclusively on the probability component. Such works encounter two problems. Firstly, they often do not reveal exactly which social groups and which individuals are at which risk of being harmed. People whose EHRs are stored in multiple databases are statistically more likely to become a victim of privacy breaches than people whose data is stored on one database. Data that is protected by insufficient security standards are more vulnerable than data that is well protected, and not anonymised breached EHRs can more easily cause harm than anonymised data [54]. The same is true for the use of medical AI. When an AI is trained and optimised with data from only one socioeconomic group or ethnicity, it is more likely for people outside this group to be harmed by biases. Secondly, even if these problems were solved and there were more accurate risk calculations available, those calculations cannot tell whether the quality of a given harm is reasonable to bear for every individual, for certain social groups, or for society. Therefore, I think, it is worthwhile to pay more attention to the quality of harms that may occur in the EHR sharing process or through the use of medical AI.

The individual risks entailed by EHR sharing are hacker attacks, leaks, and instances of data misuse that can harm individuals in multiple ways. Patients can experience psychological stress when their health-related information is leaked and becomes public [55]. They can suffer economic losses when their data are hacked and used for blackmail. They can suffer a loss of autonomy when their data are misused to support political causes or social changes without their consent [26]. Patients who do not believe these risks to be reasonable may develop trust issues regarding all medical procedures in which EHRs are generated. A real-life example of this apprehension is the failure of the NHS project ‘care.data’, which tried to extract GP surgery data into a central database that was supposed to support research, public health planning, and commercial use. Patients were allowed to opt out of this program, but information concerning how to do so was not communicated transparently. This situation caused a significant number of patients to avoid seeking medical help and to stop disclosing relevant medical information to their physicians. Eventually, the project was paused a year after its launch in 2013 due to massive protests [53, 56].

As the NHS case shows, trust and confidentiality issues are major social risks. Social risks can manifest in a decay of solidarity, instances of discrimination, and even tendencies towards human rights violations. The effects of a decay in solidarity can occur when EHRs are linked to other personal profiles. For example, insurance companies can use health data to individualise risk categories and calculate premiums [57–60], and drug companies can target patient data to drive up prices and prescriptions [61]. Governments and health insurance companies can also discriminate against other market agents by granting EHR access to exclusive business partners without communicating their cooperation transparently and without enabling citizens to withdraw from data sharing policies. For example, in 2015, the British NHS granted *Google’s Deep Mind* exclusive access to 1.6 million health records, and in 2018, the US health care provider *Ascension* made the non-anonymised health data of more than 50 million individuals available to *Google* [62]. Another type of discrimination can occur in the employment context. Leaked health information concerning employees can put employers in a position to build up discriminatory health-related hiring barriers [63]. As the two law experts Price and Cohen put it, even if there are a number of laws in the US and Europe that prohibit discriminative hiring practices (e.g., the Americans with Disabilities Act), “they can be hard to enforce because it is often hard to know when discrimination has occurred” [64].

When a collection of medical data is comprehensive enough to include a large portion of a society and when it contains sensitive information, there is also a real risk of human rights violations. Data collections can be used to identify and discriminate against social groups with certain medical or genetic characteristics that are viewed as undesirable or deleterious by political authorities. As the historians Seltzer and Anderson have shown, data items most commonly used to target populations in the past included ethnicity, religion, country of birth, and native language [65]. Since macro-political shifts and revolutions are rarely predictable events, comprehensive health databases that are collected in democracies today might support totalitarian regimes in committing human rights violations in the future [52].

There are also harms related to the use of medical AI. On a personal level, people can be harmed by erroneous medical AI. An AI causes errors if it interprets data incorrectly, generates false outputs, makes harmful therapeutic suggestions, or physically harms people due to a malfunction. An example for such problems is *the IBM* supercomputer *Watson*, which was reported to suggest unsafe and incorrect cancer treatments in a cancer research trial [66]. Errors can also be a product of human intentions. These so-called *adversarial attacks* can be introduced to any learning algorithm [67]. For example, it is possible to manipulate medical images with pixel noise in such a way that it is invisible to the human eye and that image recognition software will misdiagnose the images [68]. In addition to intended and unintended technical errors, patients may face trust issues regarding changes in patient–physician relationships. These changes can be caused by the concern that caregivers might be less skilled in contexts where key medical competences are performed by medical AI in the future [69, 70]. The fear of social isolation caused by the replacement of human interactions with socially engaging AI (e.g., chat bots) is also a part of this picture. Blasimme and Vayena summarise these problems as follows: “exclusive reliance on algorithms may rule out that necessary degree of flexibility that allows healthcare operators to calibrate objective criteria with the reality of each individual case” [71].

The use of medical AI also entails the social risks of a decay of solidarity. If medical AI is going to be as effective as predicted, patients may feel that, in many ways, their life depends on the tech companies that own the best medical AI [72]. If those developers of medical AI increase prices or block the transfer of knowledge, solidarity structures might break apart [73]. Finally, the possibility of using medical AI to connect anonymized health data with non-anonymised datasets may facilitate human rights violations [14–16]. For example, Wang and Kosinski built a deep neural network that used a database of 35,326 facial images of self-reported homosexual men and women living in the US to learn how to recognise facial expressions that are characteristic of gay men and women [74]. It is not difficult to imagine situations in which tools that can recognise correlations among health information, social features and a person’s appearance could be used by political authorities to target citizens and undermine democratic structures [75].

Despite all these potential harms, advocates of P2 may still make the pragmatic argument that unwillingness to engage in medical data research and medical AI development projects can produce a *selection* and a *consent bias* that reduces the quality of medical AI. That is, in hypothetical comparison between a world with a civic duty to share EHRs and a world without such a duty, the first one would have relatively fewer biases and, thus, be more beneficial [24]. Given the tremendous number of other factors that also affect the quality of medical AI outputs, however, this argument would be short-sighted at best. Erroneous and, thus, potentially discriminatory and harmful AI outputs may result from errors in data transfer, from incorrectly coded diagnoses and therapies, or from incomplete and insincere patient testimonies [63]. In addition, there are other types of biases that cannot be attributed to citizens’ willingness to support medical AI development [42, 43]. There can be *capture bias* when training data are preselected according to the preferences of users, physicians, or developers. Those preferences may ignore the needs of certain social groups. There can also be a *negative* or a *positive set bias* when the control data are selected so poorly that the medical AI produces false negatives or false positives for certain groups of people. There can be an *automation bias* in the process of AI use, which is caused by the empirical fact that caregivers are less likely to question algorithmically generated diagnostic results [76, 77]. In addition, it is difficult or even impossible for patients and health care professionals to understand how complex algorithms work, whether an output is erroneous, or how a given error came about. That is why non-explainable algorithms are also known as *black box* algorithms [55, 78]. All these factors can lead to poorer outcomes for some individuals and social groups compared to a world without medical AI [79].

Overall, there are a multitude of potential risks that are important in the medical AI development process and in the use of such AI. AI projects that can be considered low risk and high benefit and that, therefore, might inspire a civic duty to share EHRs need to prove their low risk profile by (i) compiling databases and collecting datasets in a way that makes re-identification improbable, (ii) constantly updating cyber security standards, (iii) not accessing data items that are not necessary for the development process, (iv) not developing medical AI that can easily be used to discriminate or endanger individuals or social groups. However, because these characteristics apply only to very few medical AI development projects, the ‘low risks, high benefits argument’ has very limited potential to establish a civic duty that obviates IC requirements.

#### Why medical AI is not necessarily beneficial for society

It is doubtful that the benefits of medical AI can be enjoyed by every citizen. Analogous to many other medical innovations, it is more likely that in many countries, market access barriers will benefit only rich and privileged patients and those who sell medical AI [80]. Some authors try to counter this view by introducing the economic trickle-down effect to the public health sphere. Ballantyne and Schaefer, for example, argue that new medical innovations might be very expensive at first and only accessible to the wealthy. However, just as the wealth of the rich will trickle down through all social classes, eventually, the positive effects of innovative medicine will benefit everyone in a society [22]. Apart from the trickle-down effect is more of an economic thesis than an empirically proven phenomenon [81], some critics like Benke and Benke believe that medical AI innovations contribute to the issue of unequal access to health care in most countries [67, 68]. Advocates of premise P3 may still reply that even if economic or location-based disadvantages might exclude certain people from innovative health care techniques, the development of new and innovative health care technology including medical AI will, nonetheless, consolidate existing medical knowledge [21] and increase the common good [22]. This counterargument is problematic for the same reasons. Mechanisms such as paywalls and intellectual property rights can shift benefits into private spheres, and digital and medical literacy issues as well as discriminatory infrastructure can deny certain social groups access to medical knowledge [64].

In light of these arguments, a duty to share EHRs that equally applies to all citizens and that may even bypass IC requirements appears to be rather unfair in all cases in which citizens do not enjoy equal access rights to medical AI products [65]. Therefore, it can be concluded that P3 is justified only if (v) the medical AI in question will most likely be beneficial to all citizens despite socioeconomic differences, if (vi) the data use and the development process are communicated transparently, and if (vii) the dataset cannot be reused for other purposes without IC.

### The ‘property rights argument’

It is highly controversial in European and US law, whether EHRs can or should be seen as something a person can have property rights over [82, 84]. Although I will not discuss this complex legal matter here, I will, nonetheless, refer to the following rationale as ‘property rights argument’. I do so because the scholars presented in this section focus on various concepts of ownership and the exclusive claims individuals, institutions, and companies should or should not have on EHRs. Most of them do so from an ethical perspective and without reference to established laws. The ‘property rights argument’ can take two forms. Firstly, in the spirit of John Locke’s concept of property, it is argued that institutions co-create the value of health data collections by collecting, digitising, and organising data in health care and research processes. Without tremendous administrative and financial investments, there would not be anything valuable to which exclusive ownership claims could be made. Therefore, public institutions that generate health data collections are entitled to use that data in the public’s best interest. Citizens might have a legitimate interest in privacy but they cannot claim exclusive rights on data about their health [27, 72, 73]. This narrative is often accompanied by the taxpayer analogy [30, 35, 75, 83], which argues as follows: in most countries, citizens whose earnings are above a minimal threshold must pay income taxes. This tax money can be seen as a contribution to the common good. Citizens have a duty to pay their taxes, and the taxes are used in a way governmental institutions see fit. Even though the money can be used on projects that certain taxpayers do not wish to support (e.g., the military budget, abortion clinics, churches), paying taxes is considered a fair social practice that involves everyone doing her or his part. Transferred to the context of medical research, the analogy suggests that EHRs should be seen as a fair contribution to society that should be collected and treated in the way that a given governmental institution sees fit.

Cohen presents an alternative second approach. By recalling old work cases, health care professionals such as physicians and nurses collect “little data” concerning every patient they have encountered, and they use this information to improve their skills. This “little data” collection is widely considered the property of the health care professional and not of the individual patients to whom the data pertain. Analogously, there is no reason to think otherwise when it comes to big data in health care. In both cases, the data are generated as a by-product of health care, and as such, they belong to the system or agent that provides such care [19]. Again, none of the scholars cited mentioned medical AI specifically. Nevertheless, as part of medical research medical AI can be considered here. The ‘property rights argument’ can be summarised as follows:

1. The value of many health data collections is generated by health care and research processes that are administered and financed by governmental institutions.
2. By engaging in the process of health data creation, agents gain rights over these data.
3. Governmental institutions have the right to use health data collections in the best interest of all citizens. That may include the development of medical AI.

#### Why citizen do not lose their rights to EHRs

I see at least five issues with the ‘property rights argument’. Firstly, there is the issue of co-production of EHRs. Montgomery argues that the raw data, EHRs are made of, are not generated by the health care system but by the patient’s body [85]. Thus, Montgomery concludes, that the role of individuals as co-producers of valuable data and the importance of EHRs to their autonomy make it difficult to grant others exclusive rights over EHRs and restrict IC rights. Secondly, there is the socio-economic issue that, without further adjustments, the ‘property rights argument’ would grant not only governmental institutions but also private companies the right to own and use health data collections in a way that they believe would serve the citizen’s best interest. Big tech corporations such as Google or 23andMe collect and manage health-related data from corporate trial series and consumer experiences at great financial and administrative expense. They can use these data collections for medical AI development, and they can sincerely believe that their enterprise is in the best interest of all. One way to avoid this problem, I think, would be to emphasise the data subjects’ rights as citizens to political participation. If data subjects would be able to engage in a democratic process that determines what a beneficial medical AI should look like, this process could grant and revoke governmental and non-governmental medical AI development projects a social license to operate [53, 86].

Thirdly, scholars have discussed the pragmatic issue that governmental ownership claims to EHRs might be perceived as a violation of confidentiality [53, 56]. As it has been shown in the ‘care-data’ case, confidentiality and trust are paramount in health care contexts. If citizens in their role as patients, consumers, and research subjects feel as if they do not have a say in how their health data are used, they might avoid its co-production. Fourthly, there is an ontological issue with the taxpayer analogy. Ploug argues that while the misuse of tax money does not (directly) effect taxpayers’ ability to shape their personality, the misuse of health data can violate citizens’ autonomy. Additionally, in cases where tax money has been misused, it is often possible to pay back the money and compensate for damages. Leaked information concerning a real person’s health, however, cannot be taken back [63]. I am going to criticise Cohen’s comparison between the memory of professional caregivers and health databases in the same way. Physicians cannot tell if people they encounter in their daily lives match cases from textbooks or colleagues’ reports. In contrast to the data in the mind of a person, EHRs can be accessed by multiple agents and used outside the confidential patient-caregiver relationship. In addition, certain types of medical AI and certain institutions are able to combine different datasets and re-identify data subjects. Human physicians cannot do that. Fifth and finally, I like to add the ethical issue that malicious governmental institutions in the future might abuse medical databases that are created for a beneficial purpose today. This issue becomes particularly important once sharing EHRs is understood as a fair civic contribution to the common good. If all citizens in a given society accept the taxpayer analogy, it would be logical to create health data collections that take the data of all citizens into account—an effort called for by Ballantyne [27]. As I have discussed before, this policy can promote human rights violations as soon as radical institutions emerge that can access the data or enable medical AI to do so. Given these criticisms, the ‘property rights argument’ lacks socio-economic, pragmatic, ontological, and ethical strength to justify a civic duty to share EHRs for medical AI development.

### Medical AI and the civic responsibility to share EHRs

I think that a good alternative to balance the diverse interests and values in this matter is to abandon the moral duty approach in favour of civic responsibility. Why is this change in the normative structure attractive? A responsibility approach can identify different context-sensitive courses of actions for different agents to accomplish a shared objective or live up to a shared value. It enables citizens to participate in the identification of shared values, shared objectives and correspondingly adequate actions by attributing and accepting responsibility in a reciprocal and real-life process [87]. In contrast, the duty-centred arguments presented acknowledge a comparatively small number of actions as appropriate (‘share your EHR in case X’), and they make judgements concerning the right course of action from a position that is either concerned with the risk-benefit analysis and the presumed preference of society, or from a position that tries to represent a somewhat impartial view. Such a position can anticipate values concerning medical AI, the state of future health care, privacy, autonomy, and the common good that might be shared in pluralistic societies. However, it cannot mimic or replicate the real-life process of forming, identifying, and legitimising shared values [88].

To illustrate the significance of the shift from a civic duty to a civic responsibility to share EHRs to support medical AI it is helpful to imagine a society in which such a civic duty existed. In this society, any citizen who does not want to act immoral must consent to her or his EHRs being stored and processed for medical research purposes including medical AI development. Any medical AI project like the already mentioned AI CheXNet that wants to access those data to train their AI needs to ask permission from a given authority. This authority, may it be an ethics committee or a different institution, faces the legitimacy problem described above. It can guess whether the data subjects collectively like to support the CheXNet project, or it can declare that the CheXNet AI would be in the interest of society. Either way, the authority’s decisions do not have to match the data subjects’ values nor their preferences. The gap between the data subject’s actual values and data access policies manifests when people are harmed because of this policies. That might be the case when AI projects like Packhäuser and colleagues’ deep learning algorithm that is programmed to re-identify anonymised chest X-ray images are granted access to the CheXNet training data as well and produce privacy breaches. Such cases are challenging because citizens can not be addressed as responsible agents properly here. They are not sufficiently responsible for the harms that might be caused by the privacy breaches because of two reasons. Firstly, they are not in a position in which they co-create the structural conditions of medical research and medical AI development. Secondly, they are also not in a position in which they can form shared ideas of eligible and undesirable research projects. Because they have already done their duty and obeyed the moral imperative to share their EHRs, it is hardly possible to motivate them to take on responsibility for the consequences of EHRs access and process policies they had no say in.

Given these problems, I think a responsibility approach is more promising. Entertaining a responsibility approach, citizens can engage in a discourse about which medical AI development projects should be supported and which not. The results of such discourses can indicate a responsibility to support those development projects and they can also motivate citizens take on this responsibility by providing shared and thus understandable reasons. People in different social positions can live up to their responsibility by performing different actions. In a pandemic, for example, patients can act responsibly by sharing EHRs with AI development projects that model the future course of infection, and they can also act responsibly by not sharing their data with AI projects that can be considered unimportant or even dangerous in a pandemic. Scientists can be responsible by engaging only in medical AI projects whose characteristics conform to shared values, and they can act responsibly by engaging in research that hinders the development of unsustainable, high-risk, and/or redundant AI technologies. Companies can act responsibly by respecting data protection laws and members of governmental institutions can act responsibly by establishing and maintaining safeguards for EHR transfer, access, and reuse. Companies and governments can also be responsible by engaging in public dialogue and promoting important medical AI developments.

The responsibility approach can be implemented with the help of modern IC models. Modern IC models such as the ‘dynamic consent model’ [89], the ‘meta-consent model’ [90], or the ‘value-based consent model’ [91] enable citizens to manage their consent decisions for secondary data use. Citizens can use such models to enter into dialogue with researchers, read additional information about medical AI projects that might increase the awareness of the benefits and risks involved in a project, ask follow-up questions, weight arguments and give their consent. They can also use the models to revoke consent decisions. With every new research request send to them, they gain the opportunity to take on responsibility – for the state of medical research, including medical AI, and also for the positive and negative consequences of that development.

## Conclusion

The contemporary arguments in favour of a civic duty to support certain medical AI develop-ments by sharing EHRs are based on premises that hardly reflect real-life research contexts. In reality, the number of electronic health data items that are generated, collected, and stored in everyday medical and non-medical encounters is increasing, as is the number of hacker attacks, health data leaks, and progress in re-identification techniques. In reality, there are numerous non-minimal risks associated with the sharing of health data collections and the development and use of medical AI that can severely harm individuals, social groups, and societies. In reality, people and institutions that engage in medical AI development projects do not always intend to serve the common good but rather their own interests. These real-life facts contradict the prem-ises of all three civic duty rationales. The ‘rule to rescue’ cannot justify a duty to support medi-cal AI developments that will save lives or prevent accidents because the act of data sharing does not cause anybody to be rescued. Additionally, the rule cannot be used to determine whether the risks entailed by the development of an AI that is designed to rescue people in acci-dent-like situations are acceptable. The ‘property rights argument’ fails to legitimise a civic duty as well because it falls short on a socio-economically, pragmatically ontological, and ethical sound justification. The bundle of arguments that support the ‘low risks, high benefits’ rationale share the problem of simply ignoring potential harms while presenting many mere hypothetical benefits as given facts. With regard to the detailed analysis of risks and benefits in this article, a medical AI development project that is likely to benefit people from a medical point of view can require a civic duty to share EHRs and bypass IC rights if and only if it has the following char-acteristics: (i) the combination of data items or datasets that are used in the development process impedes re-identification techniques, (ii) data safety standards make privacy breaches improba-ble, (iii) no data items are accessed that are not necessary for the development process, (iv) the use of the medical AI in question is not likely to discriminate or endanger individuals, social groups, or nations, (v) the medical AI and the knowledge that is produced during the develop-ment process can be accessed by every citizen without any legal or economic access barriers, (vi) the data use and the development process are communicated transparently, and (vii) the dataset cannot be reused for other purposes without IC. In all other development contexts in which these characteristics are not met, there can be no moral duty to limit today’s fundamental personal rights to IC to promote the right to live in a future with relatively better health care (compared to other hypothetical futures). I believe that there are only very few medical AI pro-jects that conform to these characteristics.

I have entertained an alternative responsibility approach that is able to mediate moral values in tense social contexts. Compared to a civic duty to share EHRs the civic responsibility approach has two advantages. Firstly, the evaluation of the eligibility of medical AI projects is based on the citizen’s actual beliefs and values and not on the assessment of a committee. In this evaluation, citizens may identify good reasons to share certain EHRs for the development of certain AI projects. If that is the case then, secondly, citizens can be motivated to take on re-sponsibility in cases where medical AI projects and the processing of EHRs produce negative outcomes. In comparison, citizens are less motivated to take on responsibility for the negative consequences of their actions when those actions do not conform to personal beliefs but to so-cial or political imperatives only. One way to implement such an approach is the use of dia-logue-enabling IC models. Those models allow users to get additional information on medical AI projects, ask follow-up questions, and make justified decisions concerning which health data items they want to share, the agents with whom they want to share such data, and the types of medical AI project that should or should not be developed. Future research should draw more attention to proactive civic responsibility towards the future of medical AI and map out the real and ideal options that citizens have to express their values and to take on responsibility.

## Data Availability

Not applicable.

## Abbreviations

AI: Artificial Intelligence
EHR: Electronic Health Records
GP: General Practitioner
IC: Informed Consent
NHS: National Health Service

## Footnotes

1 There are other similar public chest X-ray databases available like the *MIT MIMIC-CXR*, which contains 371,920 images collected between 2011 and 2016, and *CheXpert*, a dataset containing 224,316 images collected and labelled between 2002 and 2017.

2 In this article, I will refer to personal electronic health records simply as “EHRs”. ‘Personal’ means that the combination of data items in the records is either identifiable or re-identifiable. This includes pseudonymised and anonymised datasets with a combination of items that can render individuals identifiable [8, 9].

3 The authors calculated the risk based on an average annual leak of 68,735 EHRs from health care providers between 2009 and 2016 in a country with approximately 318.9 million citizens. Other entities from which EHRs were leaked or stolen within this period, such as health plans or business associations, are not included in the calculation. According to the U.S. Department of Health and Human Services, 165,398,156 individuals were affected by EHR privacy breaches in the given period. That equals 20,674,156 EHR leaks annually or a risk of 6.48% of becoming a victim of her related privacy breaches. For detailed numbers, see U.S. Department of Health and Human Services online.
